# Mitigating Mutational Meltdown in Mammalian Mitochondria

**DOI:** 10.1371/journal.pbio.0060035

**Published:** 2008-02-19

**Authors:** David M Rand

## Abstract

Animal mitochondrial genomes have high rates of sequence evolution, and should decay from the accumulation of deleterious mutations. But the purging of mutant mtDNAs in a pedigree of "mutator mice" reveals the speed and power of purifying selection to maintain mitochondrial function.

Mitochondria are remarkable microorganisms. About two billion years ago, their distant free-living ancestors hooked up with a truly foreign lineage of archaebacteria and started a genomic merger that led to the most successful coevolved mutualism on the planet: the eukaryotic cell. Along the way, evolving mitochondria lost a lot of genomic baggage, entrusted their emerging hosts with their own replication, sorted out genomic conflicts by following maternal inheritance, and have mostly abstained from sex and recombination. What mitochondria did retain was a subset of genes that encode critical components of the electron transport chain and ATP synthesis enzymes that carry out oxidative phosphorylation. Because mitochondria house the biochemical machinery that requires us to breathe oxygen, it was first assumed that mitochondrial genes would show very slow rates of molecular evolution. So it was big news almost 30 years ago when mitochondrial DNA (mtDNA) evolution was observed to be quite rapid [1]. How could the genes for a highly conserved and critical function sustain the consequences of high mutation pressure and permit rapid rates of nucleotide substitution between species? Without the benefits of recombination, where offspring can carry fewer mutations than either parent, mutations should accumulate in mitochondrial genomes through the random loss of less-mutated genomes, a process referred to as Muller's ratchet [2,3]. How have mitochondria avoided a mutational meltdown, or at least significant declines in fitness?

In many ways, these questions were set aside by researchers to capitalize on the tremendous opportunity that a rapidly evolving, nonrecombining, maternally inherited, easily sequenced set of homologous genes could provide: a window into the evolutionary history of populations and closely related species [4]. Indeed, mtDNA is still the first marker of choice for evolutionary analysis. It has played a major role in uncovering the evolutionary histories of a wide diversity of organisms, most notably our own origin and evolution from African roots [5,6]. The high rate of mtDNA evolution may have led to the assumption that most mtDNA mutations are essentially neutral and not subject to the effects of natural selection. But in recent years, there has been great interest in returning to the question of how mitochondria sustain themselves in the face of high mutation pressure and critically examining the evidence for the variety of ways that natural selection can shape the evolution of mtDNA [7–9]. It is now clear that mitochondrial mutations are a significant factor in many mitochondrial diseases [10,11]. Population and evolutionary analyses have shown that much of mtDNA variation is not consistent with the neutral model of evolution, and there is a growing debate over the relative roles of random genetic drift versus positive Darwinian and negative purifying selection in shaping mtDNA evolution [12,13].

## Mutation, Polymorphism, and Substitution Are Not the Same Thing

**Figure 1:**
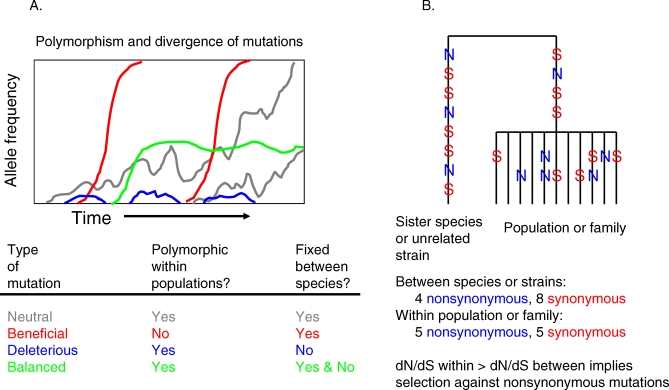
Patterns of Polymorphism for Mutations of Different Effects (A) A graph illustrating general trajectories of neutral, advantageous, deleterious, and balanced mutations. Below is a table listing the likelihood of observing these mutations as polymorphisms in a sample from within a population, or as fixed differences between species. Note that neutral, advantageous, and deleterious mutations make clearly distinct predictions. (B) An example of a McDonald-Kreitman test using the counts of nonsynonymous (N) and synonymous (S) mutations within and between species, or lineages of a pedigree. In the data shown, the dN/dS ratio is higher within species than between species, a pattern seen in many mtDNA data sets, and in the mutator mouse pedigree study reported by Stewart et al.

Rapid rates of sequence evolution can be attributed to two things: high mutation rate or low functional constraint. Inferring the actual mutation rate from patterns of sequence divergence between species, or even from variation within species, can be a problem because it makes clear assumptions about the neutrality of mutations. When mutations are strictly neutral, they have no detrimental or beneficial effects on the survivorship or reproduction of the organism, and their establishment in a population is a matter of chance (see Figure 1). Since most mutations are detrimental and are removed by selection, more mutations will be produced than are ever observed as polymorphisms within a population. Mutations that enter the population through individuals fit enough to reproduce must persist for many generations before they become fixed in the population.

In populations of finite size, weakly deleterious alleles can persist and even become fixed due to the random sampling of genetic drift. Likewise, more mutations will be observed as polymorphisms within populations than are observed as fixed substitutions between species. If mutations are generally deleterious, mutations will outnumber polymorphisms, which will outnumber fixation (substitution) events. If mutations are generally beneficial, levels of polymorphism or divergence can be higher than for deleterious mutations, but by an amount that depends on many population genetic factors (strength of selection, population size, local recombination rate, etc). Thus, one can only estimate the mutation rate from levels of polymorphism within, or substitution between, species for strictly neutral mutations. But how do we find out which mutations are neutral? And how do we estimate evolutionary time if mutation rates differ from substitution rates? At stake is the accuracy of evolutionary inference in a wide array of problems in evolutionary genetics [9,14].

There are two simple solutions to this problem: use DNA neutrality tests that compare patterns of polymorphism and fixation for nucleotide sites with different functional constraints (e.g., the McDonald-Kreitman or MK test of nonsynonymous versus synonymous mutations in protein coding regions [15]), or measure mutations in pedigrees or sets of mutation accumulation lines where selection has been weak or absent (see Figure 1). In the case of mtDNA, both of these approaches have provided evidence for selection against mildly deleterious mutations. In mitochondrial protein coding genes, the ratio of nonsynonymous/synonymous (dN/dS) polymorphisms within species is greater than this ratio between species (see Figure 1B), consistent with a purging of weakly selected amino acid–altering polymorphisms [16,17]. In pedigree studies, the mutation rate can be estimated by the frequency of new mtDNA variants observed in a sample of descendants from a founding mother. These analyses typically show that the “pedigree rate” of mutation exceeds the estimated “phylogenetic rate” of mutation by as much as a factor of 10 [18,19]. Since the “phylogenetic rates” are estimated by comparing substitutions between species on a phylogeny (or evolutionary lineage), they measure substitution, not mutation rate. Together, these data imply that mutations introduced into a pedigree are being eliminated before they become fixed within that species.

Some studies have combined these approaches by sequencing mitochondrial genomes from a set of mutation accumulation (MA) lines [20], or in lineages with very different population sizes [21]. In Caenorhabditis elegans, the dN/dS ratio among MA lines exceeds that observed among wild strains, consistent with a relaxation of purifying selection among the MA lines [20]. In the water flea, Daphnia, sexual species have larger effective population sizes than asexual lineages, resulting in weaker selection in asexual lineages. As predicted by a model of purifying selection on mtDNA, the asexual lineages show significantly larger dN/dS ratios than the sexual lineages, and analyses confirm that this is due to elevated rates of nonsynonymous evolution in the asexual lineages [21,22]. A similar effect is seen in comparisons of mtDNA divergence in large versus small mammal species, where large body size is associated with smaller population size, weaker purifying selection, and hence more rapid accumulation of deleterious mutations [23].

Again, all of these studies document that mutation does not equal polymorphism, and polymorphism does not equal fixation, consistent with the pervasive effects of purifying selection against deleterious nonsynonymous mutations. If the purging of deleterious mutations is as pervasive as it appears, these data address the question of how mitochondria can persist in the face of high mutation pressure.

## Bottlenecks and the Units of Selection Can Slow the Advance of Muller's Ratchet

But what has been lacking from these studies is a comprehensive look at the very early stages of this mutation process. When an mtDNA mutation occurs, it generates heteroplasmy, or a mixed population of mtDNAs within the cell. Because new mutations are nested in a hierarchy of populations (multiple mtDNAs within each mitochondria, multiple mitochondria per cell, many oocytes per female that may give rise to an offspring, and variable numbers of breeding females in natural populations), mutation and selection are closely connected. At any stage in the transmission though this hierarchy of populations, bottlenecks or genetic drift due to sampling of mtDNAs generate variation among lower units within higher units (e.g., mitochondria within cells, or oocytes within individual germ lines), which provides raw material for natural selection to act upon. As mutations accumulate in germline mitochondria, this variation can more effectively purge deleterious mutations than under conditions of purely clonal transmission of nonrecombining mtDNA [24]. This will delay the “ratchet” effects of mutational decline envisioned by Muller [25,26]. To get an accurate picture of how selection purges new mitochondrial mutations, we would like to document patterns of mutation, polymorphism, and fixation among units in this hierarchy.

In a recent issue of *PLoS Biology*, a new study [27] by James Stewart et al. provides a thorough analysis of how natural selection removes mutations from maternal lineages of mice and describes patterns of variation that are remarkably similar to those found in human populations. The data provide a very clear picture of purifying selection in action. The researchers took advantage of a knock-in mouse model that carries a mutation for the proofreading activity of the mitochondrial DNA polymerase, polymerase gamma (*polgA^mut^/polgA^mut^*) [28,29]. These mice have elevated mtDNA mutation rates, accelerated senescence, and a number of phenotypes associated with mitochondrial diseases. Mice homozygous for this mutation were bred to a standard lab strain to generate homozygous wild-type F2 offspring with normal mtDNA polymerase function (*polgA^+^/polgA^+^*). Because these F2 mice descended from the homozygous mutant mother, they carried a mixed population of mutant mtDNAs in their germ line and somatic tissues.

Stewart et al. sequenced the complete mtDNAs from 190 mice in the pedigree, from F2–F6. Many more mutations were observed in the third codon positions of the protein coding genes, consistent with selection against amino acid–altering mutations that are more common in first and second codon positions. Earlier work on a small sample of homozygous mutant mice showed that initial mutations were equally likely to occur at all codon positions [28,29], which should generate a dN/dS value of approximately 1.0. The overall dN/dS ratio was 0.6035, and this value dropped to 0.4617 when mutations observed in single mice were excluded. Thus, rare mutations were more likely to be nonsynonymous and were purged, as expected under purifying selection. The dN/dS ratio was significantly higher among the mutator strain pedigree mice than that for polymorphism among 21 wild-type strains of mice, or for divergence to a different species, Mus musculus molossinus. Stewart et al. further show that the observed mutations at 4-fold degenerate codon positions (those that can mutate to any nucleotide without altering the encoded amino acid and hence are very close to neutral) are significantly more homogeneous across mitochondrial genes than non-4-fold degenerate sites, suggesting more selection on functionally constrained nucleotide changes. This rate differential was most pronounced for the cytochrome oxidase subunits, which are the most evolutionary conserved mitochondrial proteins.

The observed patterns of nonsynonymous and synonymous changes across the first, second, and third codon positions of protein coding genes in the mutator mouse data were evident by the F2 generation, suggesting that selection to remove amino acid changes occurs very rapidly in mouse pedigrees. Notably, these patterns of variation across codon positions were remarkably similar to those for a sample of complete human mtDNAs, implying that most of the purging of deleterious mutations in mammals occurs in the few generations after the mutations are introduced.

For transfer RNA, ribosomal RNA genes, and the noncoding D-loop, the patterns of mutation observed in the mutator mouse data were curiously different than those for wild-type mouse strains or humans. In most animals, variation in mitochondrial tRNAs and rRNAs is typically much lower than that observed in D-loop regions, but the mutator mice showed high mutation rates in tRNAs. Interestingly, tRNAs comprise less than 10% of the mtDNA coding DNA, but account for almost 60% of human pathogenic mutations. This has been taken as evidence for an important role of tRNA mutations in mitochondrial disease. But pathogenic tRNA mutations often must reach high heteroplasmic frequencies within the cell before they have pathogenic effects [11]. The new mutator mouse data suggest that negative selection on tRNAs may be rather mild. This observation warrants further study, given the common association of mt-tRNA mutations in disease.

## What Is To Be Done?

What do these results tell us about how mtDNA mutation and selection govern mitochondrial function? And how might this affect evolutionary inference using mtDNA? The strong signature of negative selection on the protein coding sequences in mtDNA, and rather limited evidence for selection on the RNA genes, suggest that the purging of deleterious mtDNA acts via selection on physiological and biochemical performance of individual mitochondria, oocytes, and possibly individual mice in these pedigrees. As any good result should do, this study raises more questions than it answers. How are the individual mutations partitioned within and among mitochondria within oocytes? Do mitochondria with more mutated mtDNAs replicate at a slower rate due to reduced oxidative phosphorylation activity? Do oocytes with more mutant mtDNAs fail in early embryogenesis at a higher rate than oocytes carrying a lower load of mutations? Is selection on mice in the pedigrees due to early selection on embryos, or to tissue-specific failures later in development?

Answers to these questions will require detailed sampling from oocytes, embryos, and neonates in larger pedigrees, but such data are as relevant to evolutionary genetics as they are to medical questions related to fertility [8,30]. It is easy to imagine how the distribution of mtDNA mutations among oocytes and embryos could provide the raw material for strong purifying selection.

If the selection against deleterious mutations is largely complete within a few generations, then what can we infer about the consistent pattern of excess amino acid polymorphism in natural populations, including humans? This pattern would suggest that most of the amino acid variants found within populations are indeed mildly deleterious, with an emphasis on mild. Nevertheless, mitochondrial mutations are a very common form of metabolic disease, so deleterious mutations are continuing to enter the human population. If excess polymorphism is a common pattern, then molecular clock approaches that infer age of the most recent common ancestor in humans will be overestimates. Methods to correct for apparent rate differences between short-time-frame and long-time-frame estimates of the most recent common ancestor have been developed [14], and certainly should be more widely applied for mtDNA [9].

The new mouse study also begs new questions about positive selection on mtDNA. The mutator mouse model [27] should certainly have generated some point mutations that are beneficial, so it is interesting that no signature of a selective sweep leading to fixation of a novel mtDNA variant was evident in the data. For such an event to have occurred, the presumed positively selected site(s) would have to have a net selection coefficient great enough to overcome the negative selection acting on mutations on the same mtDNA molecule, as well as on others in the hierarchy of populations within that pedigree. Methods are now available to estimate the distribution of fitness effects of mutations when a general pattern of negative selection is apparent that may include a mixture of advantageous and deleterious mutations [31]. Additional studies of this type are needed to provide context for recent reports of positive selection on mitochondrial genes [12,32–34].

## Abbreviations

dN/dS: nonsynonymous/synonymous
MA: mutation accumulation
